# Phase Formation Mechanism and Anomalous Magnetic Variation of High-Performance La-Co-Doped Strontium Ferrites

**DOI:** 10.3390/ma18020323

**Published:** 2025-01-13

**Authors:** Pengbo Fu, Zhenhuan Li, Fang Wang, Munan Yang, Lulu Liu, Licheng Wang, Huayang Gong, Jian Zhang, Baogen Shen

**Affiliations:** 1 Faculty of Materials Metallurgy and Chemistry, Jiangxi University of Science and Technology, Ganzhou 341000, China; f1209055893@163.com (P.F.); yangkl1930@163.com (M.Y.); 2 Ningbo Institute of Materials Technology and Engineering, Chinese Academy of Sciences, Ningbo 315201, China; lzhlink64@163.com (Z.L.); liululu99@nimte.ac.cn (L.L.); wanglichen@nimte.ac.cn (L.W.); gonghuayang@gia.cas.cn (H.G.); shenbg@iphy.ac.cn (B.S.); 3 School of Materials and Chemical Engineering, Ningbo University of Technology, Ningbo 315211, China; wangfangch@nbut.edu.cn

**Keywords:** permanent strontium ferrite, La-Co doping, phase formation mechanisms, magnetic properties, ultrapure magnetite concentrate

## Abstract

La-Co-doped ferrite is widely used due to its excellent magnetic properties, but the mechanisms of La-Co doping on its phase formation and magnetic properties remain unclear. This study clarifies the phase formation mechanisms and reveals that La-Co doping reduces the formation temperatures of the intermediate phase SrFeO_3−*x*_ and thus the final SrFe_12_O_19_ phase. This promotes complete formation of SrFe_12_O_19_, enhancing saturation magnetization. The unexpected change in coercivity after La-Co doping contradicts the variation in the determined magnetocrystalline anisotropy field. We identify that it arises from the La-Co doping lowering the formation temperature of SrFe_12_O_19_, leading to excessive particle growth.

## 1. Introduction

Ferrite magnets are currently one of the most widely used permanent magnet materials in various fields [1]. Since the discovery of hexaferrite by Philips Research Laboratories in the Netherlands [2], permanent magnet ferrites have attracted ongoing interest from researchers. The urgency to investigate these materials significantly intensified during the “rare earth crisis” of 2011 [3]. M-type strontium ferrite has captured a substantial market share due to its high corrosion resistance, a Curie temperature exceeding 700 K, a positive coercivity temperature coefficient, low cost, environmental friendliness, and insulating properties that hinder eddy currents. This material is extensively utilized in permanent magnets, magnetic recording media, telecommunications, and microwave devices [4]. As the rapid development of electric vehicles and other sectors progresses, the demand for high-performance magnets continues to increase, positioning the optimization of M-type permanent strontium ferrite as a crucial research focus.

M-type strontium ferrite belongs to the hexagonal crystal system (space group P6_3_/mmc) and features a complex crystal structure [5]. The structure of M-type strontium ferrite comprises a hexagonally close-packed arrangement formed by oxygen anions, with layers oriented perpendicular to the [001] direction [6]. In this configuration, one O²⁻ ion is substituted by Sr^2+^ for every five oxygen layers. Iron (Fe^3+^) occupies the interstices of the densely packed oxygen lattice at five distinct crystal sites: three octahedral positions (2a, 12k, 4f₂), one tetrahedral position (4f₁), and one trigonal bipyramidal position (2b) [7]. The orientation of electron spin varies across these crystal sites; specifically, the spins at 2a, 12k, and 2b are aligned upward, while those at 4f₁ and 4f₂ are aligned downward [1].

M-type hexaferrites exhibit a ferrimagnetic structure characterized by the antiparallel alignment of Fe^3+^ magnetic moments along the c-axis. This alignment arises from superexchange interactions mediated by O^2−^ ions. Taking into account the magnetic moment of the Fe^3+^ ions in each sublattice and at various temperatures, along with the site occupancy, it is found that a Fe^3+^ ion possesses a magnetic moment of 5 *μ_B_* at 0 K. Consequently, the total magnetic moment per molecule of SrFe_12_O_19_ at 0 K is calculated to be 20.6 *μ_B_* [8,9]. The various locations occupied by Fe^3+^ significantly influence the magnetic properties of M-type strontium ferrite. Consequently, optimizing the magnetic properties of the ferrite can be accomplished through ionic doping that replaces Fe^3+^ at these various sites.

As the demand for magnetic devices evolves toward smaller sizes, lighter weights, and higher performance, there is an increasing need to enhance the magnetic properties of sintered ferrite permanent magnets. To achieve improved performance in these magnets, significant research has focused on varying their composition. For example, the incorporation of rare earth elements such as La^3+^ and Ce^2+^ to replace Sr^2+^, along with the addition of transition metals such as Co^2+^, Zn^2+^, Mn^2+^, Cu^2+^, Al^3+^ and Ni^2+^ to substitute for Fe^3+^, have been explored as a means to enhance the performance of sintered ferrite permanent magnets [10,11,12,13,14,15,16,17,18,19,20,21,22]. Studies have demonstrated that doping with rare earth elements can substantially enhance the magnetic properties of M-type strontium ferrite [23]. Rare earth elements possess high atomic magnetic moments and magnetocrystalline anisotropy; the concurrent substitution of two or more elements results in a more pronounced enhancement of its magnetic properties [24]. At present, La-Co doping is widely employed in the industry as a primary method to enhance the magnetic properties of M-type strontium ferrite [14]. The co-doping of La and Co can enhance the hard magnetic properties of M-type strontium ferrite while also improving its thermal stability [10]. Currently, La-Co-doped M-type strontium ferrite is the sole commercially available doped permanent magnet ferrite from TDK and Hitachi Metals, exhibiting the highest magnetic performance. Research conducted by Iida et al. [25] reveals that when the doping concentration of La-Co (*x*) is below 0.4, the remanent magnetization slightly increases (from 4.4 kGs to 4.5 kGs), while coercivity attains its maximum value (4.8 kOe) when *x* is in the range of 0.2 to 0.4. Research by Kikuchi et al. [26] shows that La-Co doping can markedly enhance the coercivity of the magnet, although there is a slight decrease in saturated magnetization. Results from TDK’s developed ferrite products suggest that with La-Co doping, both the saturated magnetization and coercivity of the magnets exhibit significant enhancements [27]. Despite numerous investigations, the precise effects of La-Co doping on the magnetic properties of strontium ferrite and the underlying mechanisms remain not fully understood.

Research and manufacturing of strontium ferrite primarily concentrate on the preparation of strontium ferrite using red iron oxide (hematite) as the raw material. The Ruthner method, commonly employed for producing high-purity red iron oxide, inevitably generates substantial environmental pollutants, including ammonia-nitrogen wastewater discharge and silica removal sludge [28]. To mitigate the environmental pollution issues associated with the use of red iron oxide as a raw material, ultrapure magnetite concentrate (UMC)—which is environmentally friendly, has a stable chemical composition, and guarantees a reliable supply—has emerged as the preferred raw material for producing strontium ferrite. UMC is produced from natural ore via a process of “milling, magnetic separation, and reverse flotation,” which is free from pollution during production and adheres to increasingly stringent global environmental protection standards [29].

In this work, eco-friendly ultra-pure magnetite was utilized to synthesize La-Co-doped strontium ferrite Sr_1−*x*_La*_x_*Fe_11.6−*x*_Co*_x_*O_19_ (*x* = 0.05, 0.1, 0.15, 0.2), and the mechanism of M-type SrFe_12_O_19_ phase formation was investigated. Our findings indicate that La-Co doping lowers the formation temperature of the intermediate phase (SrFeO_3−*x*_), consequently reducing the formation temperature of the SrFe_12_O_19_ phase, leading to a more complete formation of SrFe_12_O_19_. This has a positive effect on the enhancement of saturation magnetization. Analysis of the saturation approach reveals that La-Co doping (*x* = 0–0.2) significantly enhances the crystalline anisotropy field of the magnet, resulting in an anticipated increase in coercivity. However, when the doping concentration *x* exceeds 0.05, the coercivity begins to decline anomalously. This is attributed to the reduced formation temperature of the SrFe_12_O_19_ phase due to La and Co doping, which results in excessive grain growth and, consequently, lower coercivity.

## 2. Materials and Methods

M-type strontium ferrite was synthesized from SrCO_3_ powder, ultrapure magnetite concentrate (UMC), La_2_O_3_, and CoO via a solid-state reaction method. The phases of the UMC material were analyzed using an X-ray diffractometer. The chemical composition of UMC was quantitatively analyzed using an inductively coupled plasma optical emission spectrometer (ICP-OES, SPECTRO ARCOS, Düsseldorf, Germany). The purity of UMC was 99.4%.

The weights of La_2_O_3_, CoO, SrCO_3_, and UMC were measured according to the theoretical chemical formula for strontium ferrite, Sr_1−*x*_La*_x_*Fe_11.6−*x*_Co*_x_*O_19_ (where *x* = 0.05, 0.1, 0.15, and 0.2). The materials were blended in a planetary ball mill at a speed of 150 rpm, with a weight ratio of balls to powder to water of 12:1:1.5; the diameter of the milling balls was 6 mm. The mixture was subsequently dried in an oven at 80 °C for 10 h to ensure complete dehydration. A suitable amount of the dried mixture was placed in a muffle furnace for sintering under the following conditions: 550 °C for 3 h, 750 °C for 3 h, and finally 1100 °C or 1200 °C for 1 h, resulting in the formation of strontium ferrite. The strontium ferrite powder was ground in an agate mortar to achieve dispersion prior to characterization. Phase analysis was performed using X-ray diffraction (XRD, D8 Advance, Bruker, Billerica, MA, USA). The samples were thermogravimetrically analyzed using a synchronous thermal analyzer (TG-DSC, STA 449F3, Selb, Germany). The microstructure was examined using a scanning electron microscope (SEM, FEI Quanta FEG 250, Hillsboro, OR, USA). Grain sizes were measured using ImageJ software. Scanning electron microscopy (SEM, FEI Quanta FEG 250, Hillsboro, OR, USA) combined with an energy dispersive X-ray spectrometer (EDS) was employed for microstructure observation and elemental analysis. Magnetic properties were measured using a physical properties measurement system (PPMS, Quantum Design, San Diego, CA, USA), applying a maximum magnetic field of ±50 kOe at 300 K.

## 3. Results and Discussion

Initially, we investigated the mechanism of the solid-state reaction between UMC and SrCO_3_ to form the M-type SrFe_12_O_19_ phase, along with the effects of La and Co doping on its formation mechanisms. The XRD results shown in Figure S1 indicate that the primary component of UMC is Fe_3_O_4._ The Fe_3_O_4_ phase contains a considerable amount of Fe^2+^. At elevated temperatures, Fe^2+^ can react with strontium oxides to produce low-melting-point impurities that may negatively impact the magnetic properties [30]. To mitigate this issue, it is crucial to first oxidize Fe_3_O_4_ into Fe_2_O_3_ at a lower temperature.

**Figure 1:**
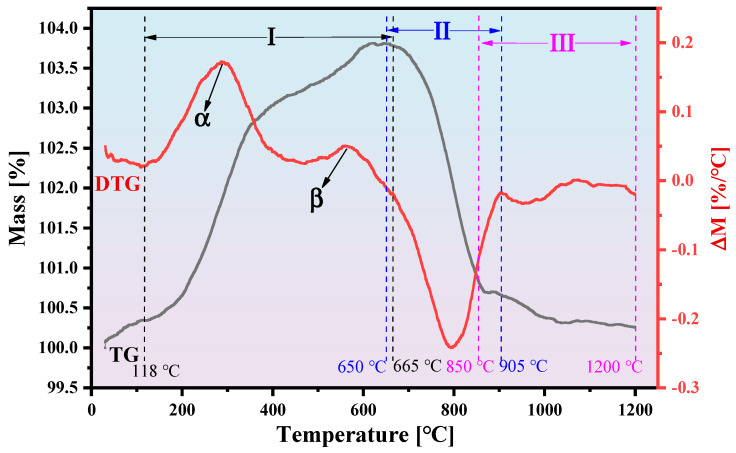
The TG and DTG curves of UMC and SrCO_3_, ranging from 30 to 1200 °C in air atmosphere. **I**: The oxidation of Fe_3_O_4_ to Fe_2_O_3_; **II**: SrCO_3_ and Fe_2_O_3_ react in the presence of air to form the SrFeO_3−*x*_ phase; **III**: SrFeO_3−*x*_ reacts with Fe_2_O_3_ to produce the SrFe_12_O_19_ phase.

To investigate the solid-state reaction process between UMC and SrCO_3_, we conducted thermogravimetric analysis on the mixture of UMC and SrCO_3_, as shown in Figure 1. The entire reaction process was divided into three stages. I: The oxidation of Fe_3_O_4_ to Fe_2_O_3_ begins at 118 °C. The mass of the sample increases with rising temperature. The first peak on the differential thermogravimetry (DTG) curve appears at point α (288 °C), indicating that the sample surface rapidly oxidizes to Fe_2_O_3_. Subsequently, as the thickness of the Fe_2_O_3_ layer increases, it hinders oxygen from penetrating the interior of the particles, resulting in a decrease in the oxidation reaction rate. When the temperature reaches point β (560 °C), the mass of the sample again increases rapidly. The high temperature accelerates the thermal motion of gas molecules, facilitating oxygen’s ability to overcome the barrier presented by the Fe_2_O_3_ layer. The thermogravimetry (TG) curve begins to decline after 665 °C, suggesting that the oxidation reaction of the sample is nearing completion. II: SrCO_3_ and Fe_2_O_3_ react in the presence of air to form the SrFeO_3−_*_x_* phase. As CO_2_ gas is generated during this process, the mass of the sample decreases rapidly, with the rate of decrease peaking at the temperature point γ (793 °C). III: SrFeO_3−*x*_ reacts with Fe_2_O_3_ to produce the SrFe_12_O_19_ phase. This reaction releases O_2_, causing a decrease in sample mass; however, the amount of O_2_ released is significantly less than the amount of CO_2_ released during the previous stage, resulting in a slower rate of mass loss [31,32]. The temperature boundaries for these three stages are not precise values but rather approximate temperature ranges. Additionally, there may be overlapping regions between the stages.

**Figure 2:**
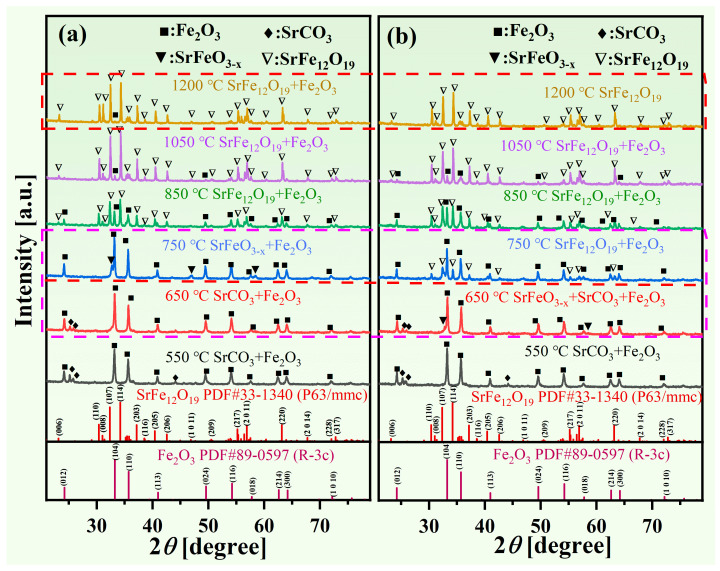
XRD patterns of Sr_1−*x*_La*_x_*Fe_11.6−*x*_Co*_x_*O_19_ samples annealed at different temperatures: (**a**) undoped La-Co; (**b**) doped with La-Co (*x* = 0.2). Pink square frame: formation temperature of the transition phase SrFeO_3−*x*_; Red square frame: Finally formed phases at 1200 °C.

To determine the formation temperatures of various phases in the solid-state reaction between UMC and SrCO_3_, XRD analysis was performed on the reaction products at different temperatures, as shown in Figure 2. The “PDF card” typically refers to the Powder Diffraction File (PDF), which is a database maintained by the International Centre for Diffraction Data (ICDD) that includes XRD diffraction patterns for various powdered samples. These cards provide essential data support for phase identification and quantitative analysis of materials. PDF#33-1340 corresponds to SrFe_12_O_19_, with a space group of P6_3_/mmc. PDF#89-0597 corresponds to Fe_2_O_3_, with a space group of R-3c. Figure 2a illustrates that after annealing at 550 °C for 3 h, Fe_3_O_4_ in the samples was fully oxidized to Fe_2_O_3_. After annealing at 750 °C for 3 h, the primary products were identified as SrFeO_3−*x*_ and Fe_2_O_3_, with no detectable SrCO_3_. This suggests that at 750 °C, SrCO_3_ reacts with Fe_2_O_3_ to form the transitional phase SrFeO_3−*x*_. At 850 °C, the SrFe_12_O_19_ phase was formed, although Fe_2_O_3_ remained present. After annealing at 1050 °C for 3 h, the amount of Fe_2_O_3_ significantly decreased, and further reduction occurred after an additional 3 h at 1200 °C.

Based on the TG-DTG and XRD results, and referring to the studies of F. Haberey and Zhou et al. [31,32], the solid-state reaction process between UMC and SrCO_3_ can be illustrated by the following reaction equations:

(1) $$550\;{}^{\circ}C:\;4Fe_{3}O_{4}+O_{2}\to 6Fe_{2}O_{3}$$

(2) $$750\;{}^{\circ}C:\;{SrCO}_{3}+\frac{1}{2}{Fe}_{2}O_{3}+\left(0.5-x\right)\frac{1}{2}O_{2}\to SrFeO_{3-x}+CO_{2}$$

(3) $$850\;{}^{\circ}C–1200\;{}^{\circ}C:\;\;SrFeO_{3-x}+5.5Fe_{2}O_{3}\to Sr{Fe}_{12}O_{19}+(0.5-x)\frac{1}{2}O_{2}$$

We conducted further investigation on the effects of La and Co doping on the phase formation mechanism during the solid-state reaction to produce the SrFe_12_O_19_ phase. Figure 2b illustrates the XRD analysis of the solid-state reaction products from UMC and SrCO_3_, doped with La and Co *x* = 0.2, following annealing at various temperatures. At 650 °C, a small amount of the SrFeO_3−*x*_ phase was detected, and its formation temperature was lower than that of samples without La and Co doping. At 750 °C, the SrFe_12_O_19_ phase emerged, and the transition phase SrFeO_3−*x*_ vanished entirely. The temperature at which the SrFe_12_O_19_ phase formed for *x* = 0.2 was nearly 100 °C lower than that of the undoped La-Co samples (see pink square frame, Figure 2). At 1200 °C, the pure SrFe_12_O_19_ phase was formed in the La-Co-doped samples, whereas the undoped samples still contained Fe_2_O_3_ impurity phase (see red square frame, Figure 2). These findings suggest that La and Co doping significantly lowered the formation temperature of the SrFe_12_O_19_ phase.

**Figure 3:**
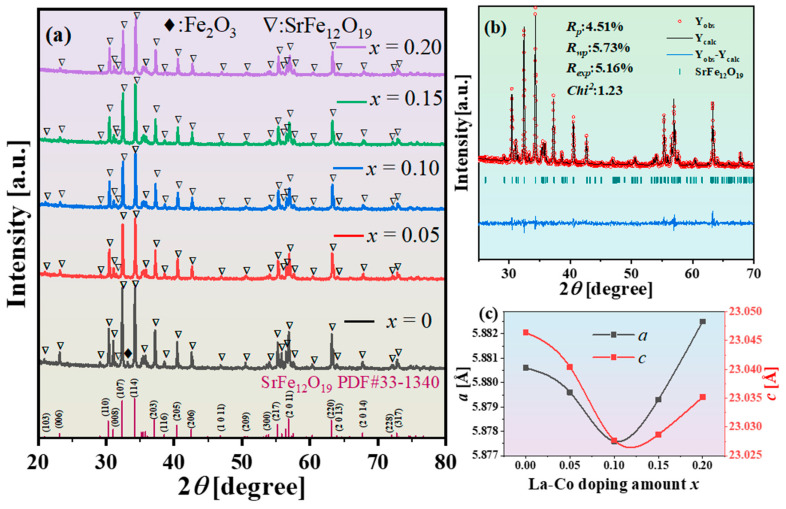
(**a**) XRD patterns of Sr_1−*x*_La*_x_*Fe_11.6−*x*_Co*_x_*O_19_ samples after sintering at temperatures of 1200 °C; (**b**) Rietveld refinement pattern of Sr_1−*x*_La*_x_*Fe_11.6−*x*_Co*_x_*O_19_ (*x* = 0.05) samples at a maximum sintering temperature of 1100 °C. (**c**) Variation trend in lattice constants of Sr_1−*x*_La*_x_*Fe_11.6−*x*_Co*_x_*O_19_ (*x* = 0–0.2) samples at a maximum sintering temperature of 1100 °C.

To investigate the effect of the La and Co doping amount on the phase composition and crystal structure of the reaction products, X-ray diffraction (XRD) analyses were conducted on the final products doped with varying amounts of La and Co. Figure 3a and Figure S2 present the XRD patterns of Sr_1−*x*_La*_x_*Fe_11.6−*x*_Co*_x_*O_19_ (*x* = 0.05, 0.1, 0.15, 0.2) samples prepared using UMC. Figure S3 presents the rietveld refinement pattern of Sr_1−*x*_La*_x_*Fe_11.6−*x*_Co*_x_*O_19_ (*x* = 0, 0.05, 0.1, 0.15, 0.2) samples at a maximum sintering temperature of 1200 °C. Table S1 presents the lattice constants and Fe_2_O_3_ content of the Sr_1−*x*_La*_x_*Fe_11.6−*x*_Co*_x_*O_19_ (*x* = 0, 0.05, 0.1, 0.15, 0.2) samples at a maximum sintering temperature of 1200 °C obtained from rietveld refinement. At a maximum sintering temperature of 1100 °C, all samples displayed SrFe_12_O_19_ phases; however, Fe_2_O_3_ impurities were also observed (see Figure S2). At a maximum sintering temperature of 1200 °C (Figure 3a), only the sample with *x* = 0 contained Fe_2_O_3_ impurities, whereas the others yielded a pure SrFe_12_O_19_ phase. This phenomenon is attributed to the doping of La and Co, which lowered the formation temperature of the SrFe_12_O_19_ phase, facilitating the complete formation of the SrFe_12_O_19_ phase at the sintering temperature of 1200 °C.

Lattice constants’ data were obtained through Rietveld refinement using FullProf software. Figure 3b shows the Rietveld refinement pattern for Sr_1−*x*_La*_x_*Fe_11.6−*x*_Co*_x_*O_19_ with doping *x* = 0.05, while Figure 3c illustrates the variation of the lattice constants of Sr_1−*x*_La*_x_*Fe_11.6−*x*_Co*_x_*O_19_ (*x* = 0.05, 0.1, 0.15, 0.2) samples after sintering at 1100 °C in relation to the La-Co doping amount. Table 1 lists the refined lattice parameters, with *R_p_*, *R_wp_*, *R_exp_*, and *Chi*^2^ being common parameters used to assess the quality of the refinement fit. The three types of *R* factors being below 10 indicate favorable data fitting results. The *c/a* ratio ranges from 3.9159 to 3.9197, which is less than 3.98, confirming that all samples in this series exhibit a magnetoplumbite structure [33]. As the La-Co doping amount increases, both the *a* and *c* lattice constants show a trend of first decreasing and then increasing. The primary reasons for the observed changes in the lattice constants of M-type strontium ferrite are as follows: (1) Substituting the larger Sr^2+^ ion (1.13 nm) with the smaller La^3+^ ion (1.06 nm) leads to a decrease in the lattice constants. (2) Replacing the smaller Fe^3+^ ion (0.64 nm) with the larger Co^2+^ ion (0.74 nm) results in an increase in the lattice constants [34]. (3) The incorporation of La^3+^ causes Fe^3+^ (0.64 nm) to transform into Fe^2+^ (0.76 nm), contributing to the increase in lattice constants [35].

**Figure 4:**
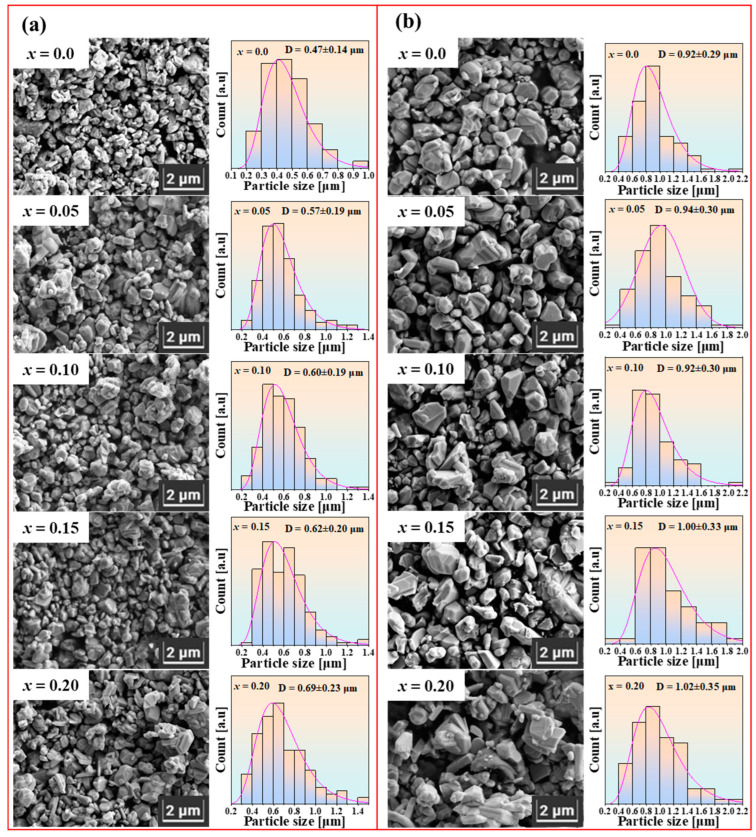
The SEM images and the particle size distribution of Sr_1−*x*_La*_x_*Fe_11.6−*x*_Co*_x_*O_19_ samples (*x* = 0–0.2) at a maximum sintering temperature of (**a**) 1100 °C; (**b**) 1200 °C.

Figure 4a depicts the microstructural morphology and particle size distribution of Sr_1−*x*_La*_x_*Fe_11.6−*x*_Co*_x_*O_19_ samples, which have been sintered at 1100 °C with varying La-Co doping amount. The particle size distribution curve is obtained by fitting a log-normal distribution model in the histogram using Origin software. Smaller particles exhibit an irregular block shape, whereas larger particles display a flaky morphology. The particle size distribution diagram shows that the average particle size increases as the doping amount of La and Co rises from 0 to 0.2. Figure 4b presents the microstructural morphology and particle size distribution of Sr_1−*x*_La*_x_*Fe_11.6−*x*_Co*_x_*O_19_ samples, which were sintered at 1200 °C with varying La-Co doping levels. For the sample with *x* = 0, the average particle size was 0.47 μm after sintering at 1100 °C and increased to 0.92 μm at 1200 °C, indicating nearly a twofold growth in size with increasing temperature. Moreover, as the amounts of La and Co doping increased, the particle size in samples sintered at 1200 °C also exhibited an obvious increase.

**Figure 5:**
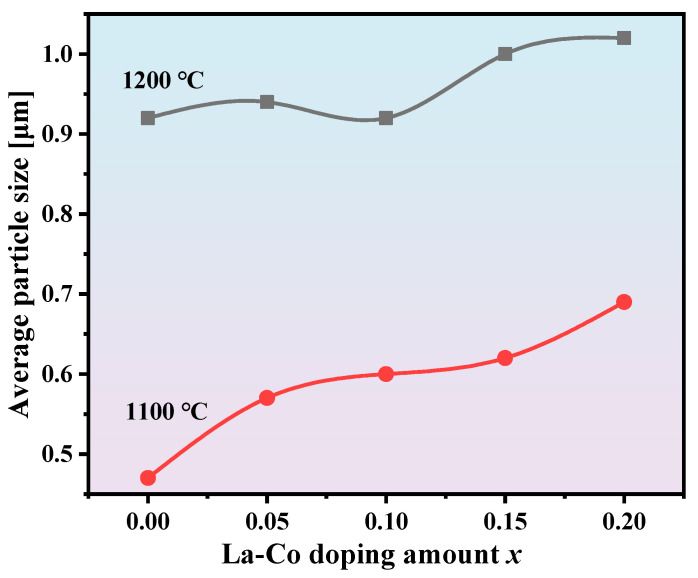
The corresponding changes in average particle size with respect to *x* at temperatures of 1100 °C and 1200 °C.

Figure 5 shows the relationship between average particle size and La-Co doping amount at sintering temperatures of 1100 °C and 1200 °C. It is clearly evident that the particle size of the samples sintered at 1200 °C is significantly larger than that of the samples sintered at 1100 °C. Additionally, as the La-Co doping amount increases, the particle size of the samples shows an increasing trend. It is well known that as temperature increases, atomic thermal motion intensifies, and diffusion rates increase, thereby promoting particle growth. At higher temperatures, grain boundaries are also more likely to migrate, and the migration of grain boundaries leads to grain consolidation, resulting in larger particle sizes. Therefore, the particle size of the samples sintered at 1200 °C is significantly larger than that of the samples sintered at 1100 °C. From the formation process of the SrFe_12_O_19_ phase (see Figure 2), it is known that La-Co doping lowers the formation temperature of the SrFe_12_O_19_ phase, which, from another perspective, is equivalent to effectively increasing the sintering temperature of the SrFe_12_O_19_ phase. Consequently, as the La-Co doping amount increases, the particle size of the sintered samples also increases.

Scanning electron microscopy with energy dispersive X-ray spectroscopy (SEM-EDS) was performed on Sr_1−*x*_La*_x_*Fe_11.6−*x*_Co*_x_*O_19_ sintered samples to check the homogeneity of element distribution (see Figure S4). For *x* = 0.05, the elements Sr, Fe, La, and Co were uniformly distributed. However, for *x* = 0.20, while Sr, Fe and Co remained uniformly distributed, slight bright spots of La were observed (see Figure S4, the red circles). This observation suggests that excessive doping of La may result in minor element segregation, potentially caused by the formation of a LaFeO_3−δ_ secondary phase due to the surplus of La doping. The low content of LaFeO_3−δ_ phase makes it difficult to detect using XRD.

**Figure 6:**
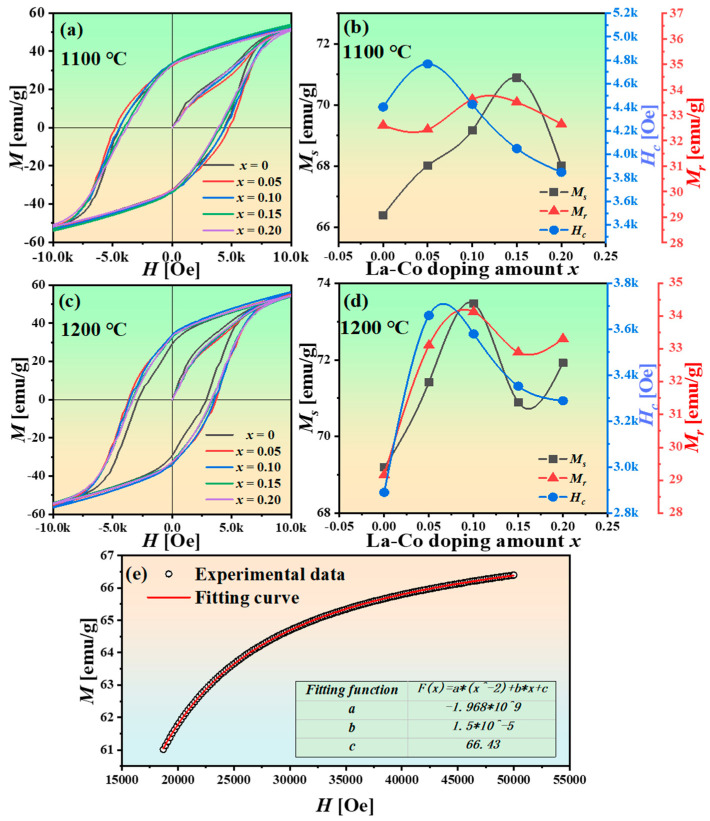
The magnetic hysteresis loops of Sr_1−*x*_La*_x_*Fe_11.6−*x*_Co*_x_*O_19_ samples (*x* = 0–0.2) sintered at 1100 °C (**a**) and the corresponding changes in magnetic properties with respect to *x* (**b**); the magnetic hysteresis loops of Sr_1−*x*_La*_x_*Fe_11.6−*x*_Co*_x_*O_19_ samples (*x* = 0–0.2) sintered at 1200 °C (**c**) and the corresponding changes in magnetic properties with respect to *x* (**d**); the fitting to the law of approach to saturation (LAS) for Sr_1−*x*_La*_x_*Fe_11.6−*x*_Co*_x_*O_19_ (*x* = 0) samples with the maximum sintering temperature of 1100 °C (**e**).

The magnetic properties of Sr_1−*x*_La*_x_*Fe_11.6−*x*_Co*_x_*O_19_ (*x* = 0–0.2) were investigated under a maximum magnetic field of ±5 kOe using a physical property measurement system (PPMS). Figure 6a–d depicts the magnetic hysteresis loops of the samples and the trends of magnetic properties as a function of the doping amount *x*, with specific values detailed in Table 2. For the Sr_1−*x*_La*_x_*Fe_11.6−*x*_Co*_x_*O_19_ samples sintered at a maximum temperature of 1100 °C, the saturation magnetization first increases and then decreases with increasing La-Co doping, reaching a peak of 70.89 emu/g at a doping amount of *x* = 0.15. Furthermore, as the La-Co doping amount increases, the coercivity initially rises and then declines, with the sample at *x* = 0.05 exhibiting the highest coercivity of 4768.84 Oe.

The squareness ratio (*R_s_*) and the Bohr magneton per unit (*n_B_*) of La-Co-doped strontium ferrite Sr_1−*x*_La*_x_*Fe_11.6−*x*_Co*_x_*O_19_ samples (*x* = 0–0.2) are estimated by the following equations [36,37]:

(4) $$R_{s}=\frac{M_{r}}{M_{s}}$$

where *M_r_* is the remanence magnetization and *M_S_* is saturation magnetization.

(5) $$n_{B}=\frac{M_{W}M_{s}\times 10^{-3}}{N_{a}\mu_{B}}$$

where *n_B_* corresponds to the magnetic moment in the Bohr magneton, *M_s_* for the saturation magnetization (emu/g), *M_W_* is the sample’s molecular weight (g/mol), *N_a_* is the Avogadro’s number (6.023 × 10^23^ mol^−1^), *µ_B_* is the electron magnetic moment (9.27 × 10^−24^ A·m^2^).

The squareness ratio (*R_s_*) indicates the strength of a material’s residual magnetism after the external magnetic field is removed. As shown in Table 2, the sample sintered at 1100 °C exhibits a higher *R_s_* than the sample sintered at 1200 °C. This can be attributed to the presence of smaller particles in the 1100 °C sintered sample, which exhibit stronger magnetic interactions, thereby enhancing the *R_s_*. In contrast, the sample sintered at 1200 °C has significantly larger particles (see Figure 4), resulting in weaker magnetic interactions and consequently a lower *R_s_* compared to the 1100 °C sintered sample. Additionally, from Table 2, it is evident that as the La-Co doping concentration increases, the Bohr magneton per unit (*n_B_*) first increases and then decreases. This suggests that a moderate amount of La-Co doping can enhance the magnetic moment of the strontium ferrite, while excessive La-Co doping diminishes the magnetic moment of the material.

The saturation magnetization and coercivity of the Sr_1−*x*_La*_x_*Fe_11.6−*x*_Co*_x_*O_19_ samples (*x* = 0–0.2) sintered at a maximum temperature of 1200 °C exhibit trends analogous to those of samples sintered at 1100 °C concerning La and Co doping. However, the saturation magnetization of the samples sintered at 1200 °C is generally greater than that of those sintered at 1100 °C. Notably, when the doping amount is *x* = 0.1, the saturation magnetization of Sr_1−*x*_La*_x_*Fe_11.6−*x*_Co*_x_*O_19_ samples attains a value of 73.47 emu/g. This enhancement is attributed to the improved phase formation of M-type strontium ferrite at elevated temperatures. In the case of M-type strontium ferrite produced through the solid-state reaction, the saturation magnetization typically increases with increasing sintering temperatures. Moreover, the coercivity of samples sintered at 1200 °C is generally lower than that of samples sintered at 1100 °C. This reduction in coercivity results from the higher sintering temperature leading to an increase in grain size, thereby decreasing the number of single-domain particles while simultaneously increasing the proportion of multi-domain particles, which exhibit lower coercivity.

To investigate the influence of La and Co doping on the magnetocrystalline anisotropy field of the M-type SrFeO_3−*x*_ phase, the saturation stage of the magnetization curves of the samples was fitted using the law of approach to saturation (LAS) [38]. As the magnetization of the samples nears saturation in the final stage, domain wall displacement becomes negligible, while the rotation of magnetic moments becomes the predominant magnetization mechanism. During this process, a substantial portion of the magnetization is dictated by magnetocrystalline anisotropy. The following empirical formula applies during the saturation magnetization phase [17]:

(6) $$M=M_{s}\left(1-\frac{A}{H}-\frac{B}{H^{2}}\right)+\chi_{P}H$$

*M_s_* represents the saturation magnetization. The term *A/H* represents magnetic inhomogeneity, arising from stray fields caused by impurities and defects, which is generally negligible at high magnetic fields. The term *B/H^2^* characterizes magnetocrystalline anisotropy, while $\chi_{P}$ denotes the paramagnetic susceptibility. For hexagonal crystal structures, *B* can be expressed as follows:

(7) $$B=\frac{H_{a}^{2}}{15}=\frac{{4K}_{1}^{2}}{{15M}_{s}^{2}}$$

*H_a_* represents the magnetocrystalline anisotropy field, while *K_1_* denotes the magnetocrystalline anisotropy constant. The magnetization curve approaching saturation (0.9*M_s_* to 1*M_s_*) was fitted using MATLAB software with Equations (6) and (7). Partial results from the fitting are shown in Figure 6e. Figure S5 presents the fitting to the law of approach to saturation (LAS) for Sr_1−*x*_La*_x_*Fe_11.6−*x*_Co*_x_*O_19_ (*x* = 0, 0.05, 0.1, 0.15, 0.2) samples sintered at 1100 °C. The fitting results demonstrate a strong correlation with the experimental data. From the fitting process, we obtained values for the fitted saturation magnetization, the magnetocrystalline anisotropy constant (*K_1_*), and the magnetocrystalline anisotropy field (*H_a_*), which are summarized in Table 3. The results indicate that the magnetocrystalline anisotropy field of the Sr_1−*x*_La*_x_*Fe_11.6−*x*_Co*_x_*O_19_ samples (*x* = 0–0.2) significantly increases with the amount of La-Co doping.

From Figure 6b,d, it is indicated that the saturation magnetization of Sr_1−*x*_La*_x_*Fe_11.6−*x*_Co*_x_*O_19_ samples (*x* = 0–0.2) initially increases before decreasing. Moderate La-Co doping enhances the saturation magnetization in Sr_1−*x*_La*_x_*Fe_11.6−*x*_Co*_x_*O_19_ samples (*x* = 0–0.2). M-type strontium ferrite displays ferromagnetic properties, with its magnetic performance affected by superexchange interactions between Fe^3+^ and O^2−^, in addition to molecular magnetic moments. At low La^3+^ doping amounts, smaller La^3+^ ions (0.106 nm) substitute for Sr^2+^ ions (0.113 nm) in the lattice, leading to lattice contraction and reduced Fe-O distances (see Figure 3c). This change strengthens the hyperfine fields at the 2a and 12k sites, enhancing superexchange interactions and thereby increasing the saturation magnetization [39]. Furthermore, Figure 2 demonstrates a decrease in the formation temperature of the transitional phase SrFeO_3−*x*_. The formation temperature for SrFeO_3−*x*_ without La-Co doping is 750 °C, compared to 650 °C for the La-Co-doped samples. At a sintering temperature of 1200 °C, undoped La-Co samples contain trace amounts of Fe_2_O_3_ as a secondary phase, while La-Co-doped samples yield a pure SrFe_12_O_19_ phase. This suggests that La and Co doping lowers the formation temperature of the SrFe_12_O_19_ phase, facilitating a more complete phase formation at the same temperature and thus increasing the saturation magnetization.

Excessive La-Co doping results in a decrease in the saturation magnetization of Sr_1−*x*_La*_x_*Fe_11.6−*x*_Co*_x_*O_19_ samples (*x* = 0.05–0.2). When excessive La^3+^ ions are integrated into the M-type strontium ferrite lattice, they convert Fe^3+^ ions into Fe^2+^ ions. On the one hand, the magnetic moment of Fe^2+^ (4 *µ_B_*) is lower than that of Fe^3+^ (5 *µ_B_*). On the other hand, the converted Fe^2+^ preferentially occupies the 4f_1_ and 2a lattice sites, weakening the exchange interactions between tetrahedral and octahedral sites. This misalignment of the spin direction of Fe^3+^ ions at octahedral sites reduces the saturation magnetization [40,41]. Furthermore, at high levels of La-Co doping, the precipitation of (Sr, La)FeO_3−δ_ phase also contributes to the decline in saturation magnetization. Although the quantity of this phase is insufficient for X-ray diffraction (XRD) detection, SEM-EDS analysis (see Figure S4) reveals slight segregation of the La element at higher doping levels. Research has demonstrated that when Co^2+^ ions enter the lattice, they occupy the 4f_1_ and 2a sites [42]. Since the magnetic moment of Co^2+^ (3 *µ_B_*) is lower than that of Fe^3+^ (5 *µ_B_*), the substitution of Fe^3+^ with Co^2+^ further diminishes superexchange interactions [43], contributing to the reduction in saturation magnetization.

From Table 3, it is evident that the magnetocrystalline anisotropy field of Sr_1−*x*_La*_x_*Fe_11.6−*x*_Co*_x_*O_19_ samples (*x* = 0–0.2) increases significantly with an increase in La-Co doping amount. However, the coercivity of Sr_1−*x*_La*_x_*Fe_11.6−*x*_Co*_x_*O_19_ samples initially increases and then decreases with varying La and Co doping levels (see Figure 6b,d). A high magnetocrystalline anisotropy field typically results in high coercivity. The decrease in coercivity with La and Co doping contradicts the increase in the magnetocrystalline anisotropy field. This anomalous decrease in coercivity may be attributed to the reduced formation temperature of the SrFe_12_O_19_ phase resulting from La and Co doping. It is well known that the coercivity of permanent magnetic materials strongly depends on their microstructure, particularly the particle size. When the particles of ferrite are small enough to allow the entire particle to behave as a single magnetic domain, these particles are referred to as single-domain particles. Since single-domain particles can only exhibit magnetization through the rotation mechanism, their intrinsic coercivity is much higher than that of multi-domain particles, which involve domain wall displacement during magnetization. For La-Co-doped samples, the SrFe_12_O_19_ phase forms at a lower temperature, potentially leading to excessive grain growth. The SEM images and particle size distribution graphs in Figure 4 demonstrate that increasing La-Co doping leads to larger grain sizes, resulting in a reduction in single-domain particle proportions and an increase in lower coercivity multi-domain particles, which consequently explains the anomalous decrease in coercivity.

## 4. Conclusions

This study utilizes ultra-pure magnetite as a raw material and employs a solid-state reaction method to synthesize high-performance La-Co-doped strontium ferrite Sr_1−*x*_La*_x_*Fe_11.6−*x*_Co*_x_*O_19_ (*x* = 0–0.2). The findings indicate that La and Co doping lowers the formation temperature of the SrFe_12_O_19_ phase, resulting in a more complete phase formation at the same temperature. As the amount of La-Co doping increases, the lattice constant first decreases and then increases. At a sintering temperature of 1200 °C, when the La-Co doping amount *x* is less than 0.10, the saturation magnetization of Sr_1−*x*_La*_x_*Fe_11.6−*x*_Co*_x_*O_19_ samples continuously increases, reaching a maximum of 73.47 emu/g at *x* = 0.10, thereby meeting the standards for high-performance permanent magnet ferrites. The enhancement in saturation magnetization can be attributed to two factors: La^3+^ doping leads to lattice contraction, and La-Co doping reduces the formation temperature of the SrFe_12_O_19_ phase, leading to more complete phase formation. Quantitative analysis using the law of approach to saturation indicates that as La-Co doping increases, the magnetocrystalline anisotropy field of Sr_1−*x*_La*_x_*Fe_11.6−*x*_Co*_x_*O_19_ samples gradually increases. For doping levels *x* < 0.05, the coercivity of Sr_1−*x*_La*_x_*Fe_11.6−*x*_Co*_x_*O_19_ samples continuously increases, reaching a maximum of 4768.84 Oe at *x* = 0.05. However, when the doping level *x* exceeds 0.05, the coercivity begins to decrease. The anomalous decrease in coercivity is attributed to La and Co doping, which lowers the formation temperature of the SrFe_12_O_19_ phase and leads to abnormal grain growth. This paper clarifies the phase formation mechanism of La-Co-doped strontium ferrite and explains the anomalous changes in the magnetic properties. The findings are significant for understanding the mechanisms by which La-Co doping affects magnetic properties and for the design and development of high-performance La-Co-doped strontium ferrites and environmentally friendly ferrite magnets.

## Figures and Tables

**Table 1:** Lattice constants of the Sr_1−*x*_La*_x_*Fe_11.6−*x*_Co*_x_*O_19_ (*x* = 0–0.2) samples at a maximum sintering temperature of 1100 °C obtained from Rietveld refinement.

Doping Amount *x*	*a* [Å]	*c* [Å]	*c/a*	*R_p_*	*R_wp_*	*R_exp_*	*Chi* ^2^
0	5.8806	23.0464	3.9191	4.63	5.86	5.39	1.18
0.05	5.8796	23.0404	3.9197	4.51	5.73	5.16	1.23
0.10	5.8776	23.0277	3.9179	4.77	5.97	5.22	1.31
0.15	5.8793	23.0287	3.9169	4.66	5.87	5.17	1.29
0.20	5.8825	23.0352	3.9159	4.81	6.06	5.14	1.39

**Table 2:** The magnetic properties of Sr_1−*x*_La*_x_*Fe_11.6−*x*_Co*_x_*O_19_ (*x* = 0–0.2).

Sintering Temperature [°C]	Doping Amount *x*	*M_s_* [emu/g]	*M_r_* [emu/g]	*H_c_* [Oe]	*R_s_*	*n_B_*
1100	0	66.40	32.60	4402.36	0.49	12.36
1100	0.05	68.02	32.44	4768.84	0.48	12.70
1100	0.10	69.17	33.62	4425.21	0.49	12.95
1100	0.15	70.89	33.51	4048.99	0.47	13.30
1100	0.20	68.01	32.65	3847.99	0.48	12.79
1200	0	69.19	29.16	2890.07	0.42	12.88
1200	0.05	71.42	33.11	3660.09	0.46	13.33
1200	0.10	73.47	34.13	3580.02	0.46	13.75
1200	0.15	70.89	32.90	3352.44	0.46	13.30
1200	0.20	71.92	33.31	3288.77	0.46	13.53

**Table 3:** Fitted saturation magnetization *M_s_*, magnetic crystal anisotropy constant *K_1_*, and magnetic crystal anisotropy field *H_a_* of the samples at a maximum sintering temperature of 1100 °C.

Doping Amount *x*	Fitted *M_s_* [emu/g]	*K_1_* [×10^6^ erg/cm^3^]	*H_a_* [kOe]
0	66.43	3.57	21.08
0.05	68.42	3.94	22.72
0.10	69.50	4.07	23.07
0.15	71.36	4.34	24.01
0.20	68.46	4.24	24.45

## Data Availability

Data are contained within the article and Supplementary Materials.

## Supplementary Materials

The following supporting information can be downloaded at: https://www.mdpi.com/article/10.3390/ma18020323/s1, Figure S1: XRD pattern of UMC; Figure S2: XRD patterns of Sr_1−*x*_La*_x_*Fe_11.6−*x*_Co*_x_*O_19_ after sintering at 1100 °C; Figure S3. Rietveld refinement pattern of Sr_1−*x*_La*_x_*Fe_11.6−*x*_Co*_x_*O_19_ samples at a maximum sintering temperature of 1200 °C. (a) *x* = 0; (b) *x* = 0.05; (c) *x* = 0.10; (d) *x* = 0.15; (e) *x* = 0.20; Table S1: Lattice constants and Fe_2_O_3_ content of the Sr1-*x*La*x*Fe11.6-*x*Co*x*O19 (*x* = 0–0.2) samples at a maximum sintering temperature of 1200 °C obtained from Rietveld refinement; Figure S4: The SEM-EDS images of Sr_1−*x*_La*_x_*Fe_11.6−*x*_Co*_x_*O_19_ (*x* = 0–0.2) samples after sintering at 1100 °C for (a) *x* = 0.05; (b) *x* = 0.2. The red circle indicates the microsegregation of element La; Figure S5: Fitting to the law of approach to saturation (LAS) for Sr_1−*x*_La*_x_*Fe_11.6−*x*_Co*_x_*O_19_ (*x* = 0–0.2) samples sintered at 1100 °C: (a) *x* = 0.0; (b) *x* = 0.05; (c) *x* = 0.10; (d) *x* = 0.15; (e) *x* = 0.20.
