# Supplementary material for: Phase Formation Mechanism and Anomalous Magnetic Variation of High-Performance La-Co-Doped Strontium Ferrites
Source: Materials (Basel). 2025 Jan 13;18(2):323. doi: 10.3390/ma18020323 (PMC11766773; doi:10.3390/ma18020323)
Supplement: Supplementary file 1 [file materials-18-00323-s001.zip › materials-3413233-supplementary.pdf]

materials

MDPI

Supplementary Material

Phase Formation Mechanism and Anomalous Magnetic Variation of High-Performance La-Co-Doped Strontium Ferrites

Pengbo Fu[1,2], Zhenhuan Li[1], Fang Wang[3], Munan Yang[1], LuLu Liu[2], Licheng Wang[2], Huayang Gong[2], Jian Zhang[2,*], Baogen Shen[2]

This file includes:

Figure S1. XRD pattern of ultrapure magnetite concentrate (UMC).

Figure S2. XRD patterns of $Sr_{1-x}La_xFe_{11.6-x}Co_xO_{19}$ after sintering at 1100 °C.

Figure S3. Rietveld refinement pattern of $Sr_{1-x}La_xFe_{11.6-x}Co_xO_{19}$ samples at a maximum sintering temperature of 1200 °C. (a)x = 0; (b)x = 0.05; (c)x = 0.10; (d)x = 0.15; (e)x = 0.20;

Table S1. Lattice constants and Fe_2O_3 content of the Sr1-xLaxFe11.6-xCoxO19 (x = 0 - 0.2) samples at a maximum sintering temperature of 1200 ° C obtained from Rietveld refinement

Figure S4. The SEM-EDS images of $Sr_{1-x}La_xFe_{11.6-x}Co_xO_{19}$ (x = 0 - 0.2) samples after sintering at 1100 °C

Figure S5. Fitting to the law of approach to saturation (LAS) for $Sr_{1-x}La_xFe_{11.6-x}Co_xO_{19}$ (x = 0 - 0.2) samples sintered at 1100 °C

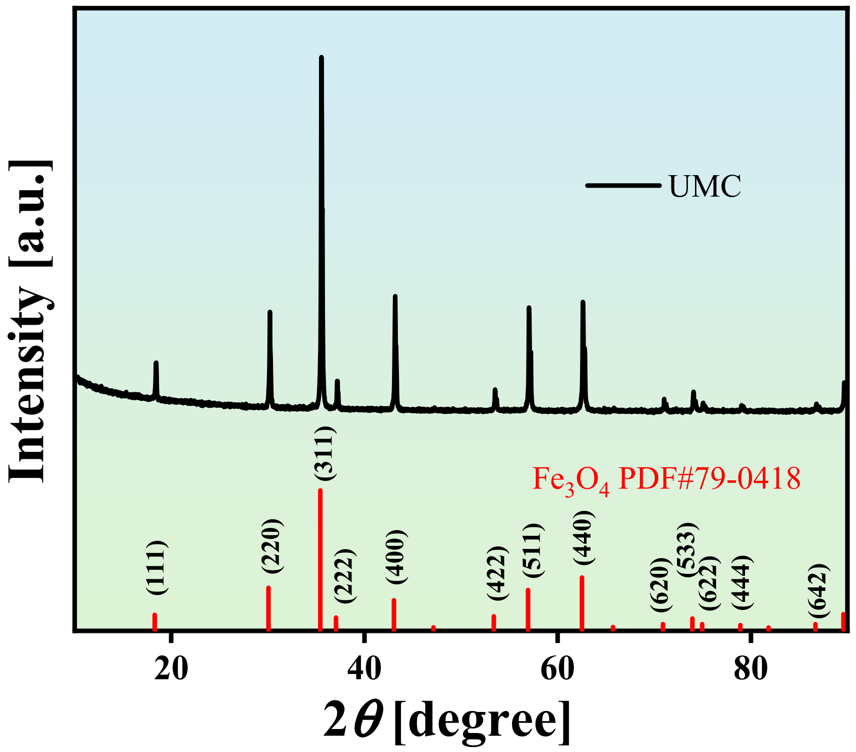

Figure S1. XRD pattern of UMC.

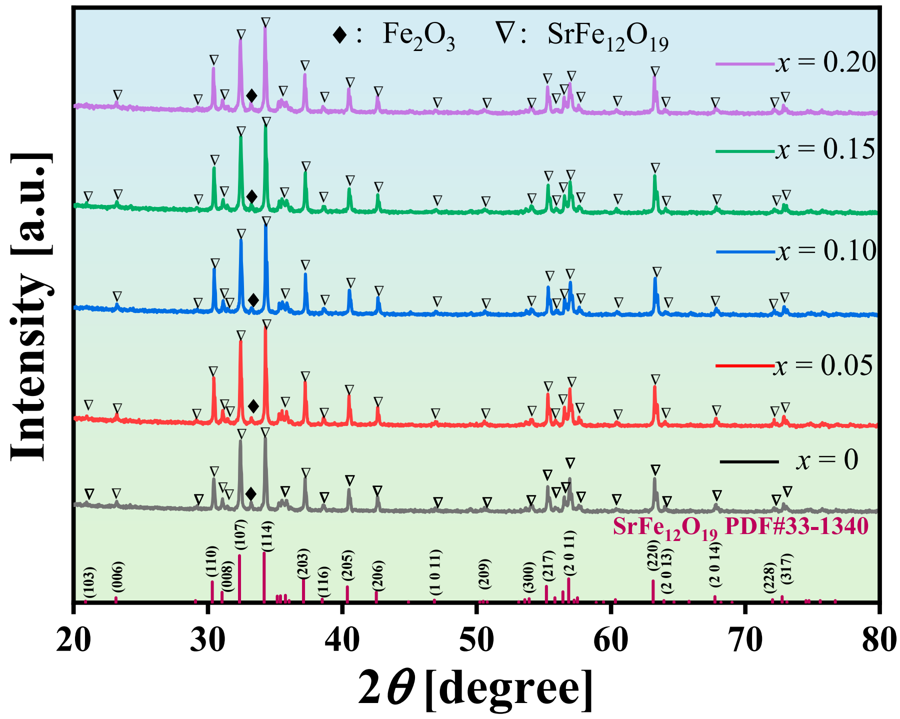

Figure S2. XRD patterns of $Sr_{1-x}La_xFe_{11.6-x}Co_xO_{19}$ after sintering at 1100 °C.

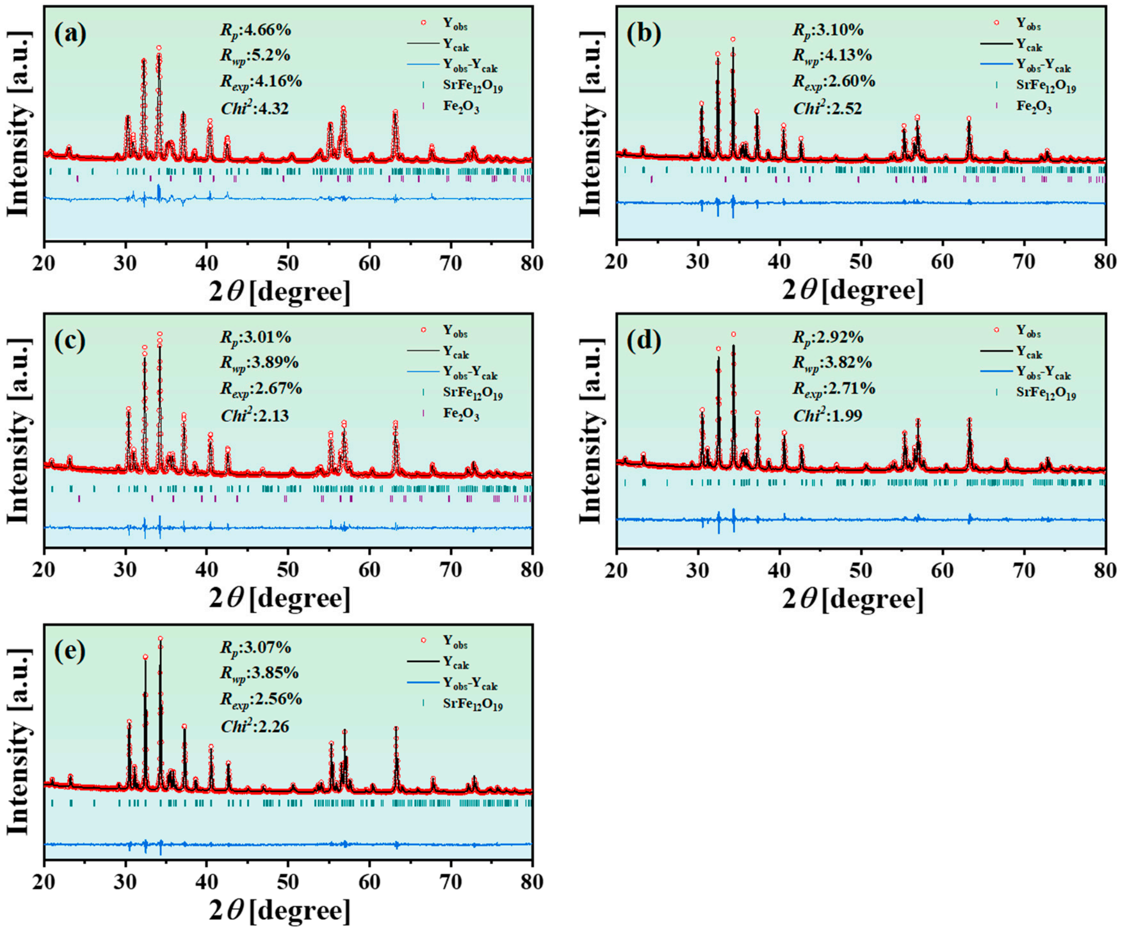

12

Figure S3. Rietveld refinement pattern of $Sr_{1-x}La_xFe_{11.6-x}Co_xO_{19}$ samples at a maximum sintering temperature of 1200 °C. (a)$x = 0$; (b)$x = 0.05$; (c)$x = 0.10$; (d)$x = 0.15$; (e)$x = 0.20$;

Table S1. Lattice constants and Fe_2O_3 content of the Sr1-xLaxFe11.6-xCoxO19 (x = 0 - 0.2) samples at a maximum sintering temperature of 1200 ° C obtained from Rietveld refinement

Doping amount *x*	*a* [Å]	*c* [Å]	*c*/*a*	Fe_2O_3 content [%]
0	5.8763	23.0404	3.9209	1.18
0.05	5.8751	23.0390	3.9214	0.32
0.10	5.8767	23.0409	3.9207	0.24
0.15	5.8741	23.0311	3.9207	0
0.20	5.8772	23.0439	3.9208	0

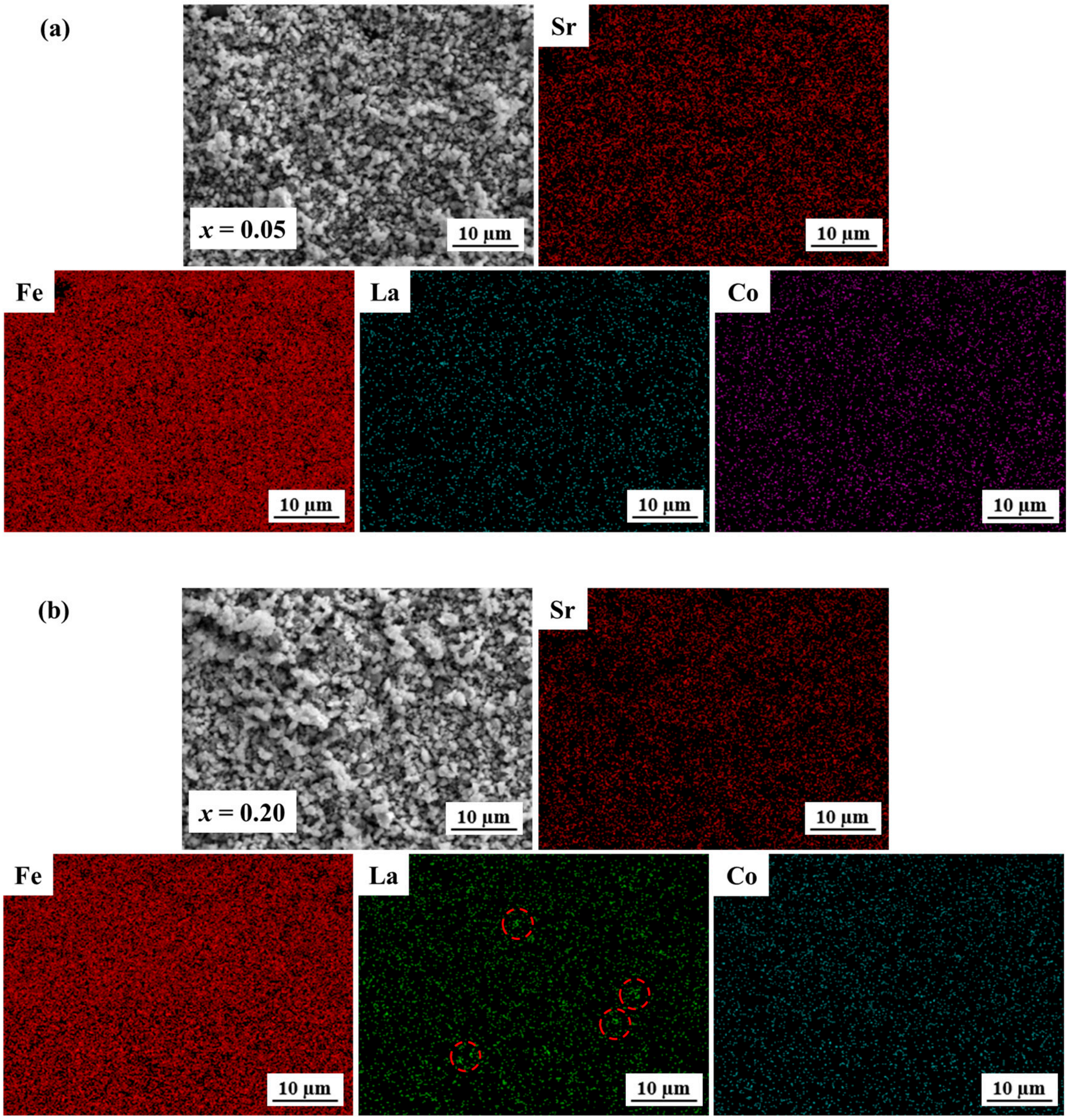

Figure S4. The SEM-EDS images of $Sr_{1-x}La_xFe_{11.6-x}Co_xO_{19}$ (x = 0 - 0.2) samples after sintering at 1100 °C for: (a) x=0.05; (b) x=0.2. The red circle indicates the microsegregation of element La.

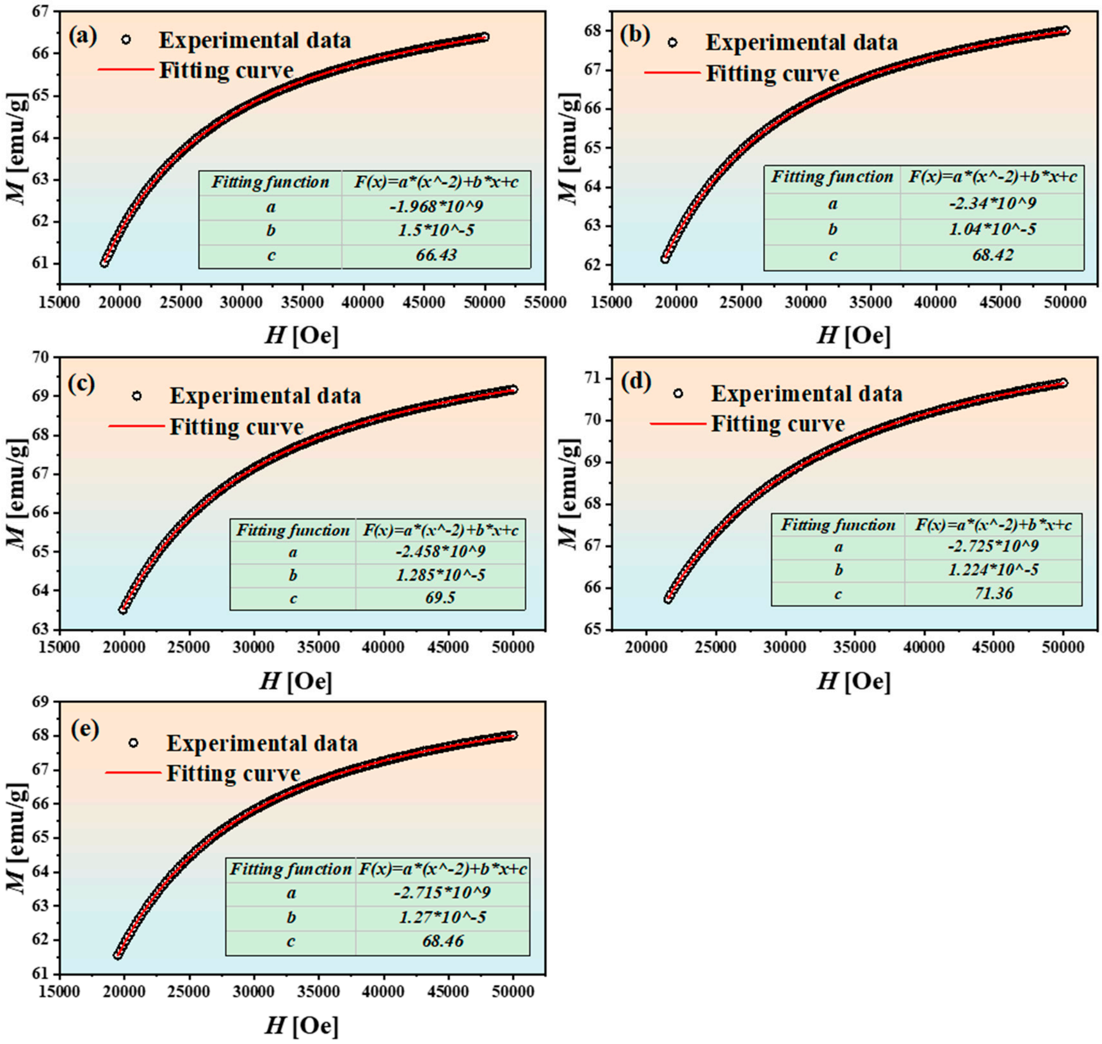

Figure S5. Fitting to the law of approach to saturation (LAS) for $Sr_{1-x}La_xFe_{11.6-x}Co_xO_{19}$ (x=0-0.2) samples sintered at 1100 °C: (a) x=0.0; (b) x=0.05; (c) x=0.10; (d) x=0.15; (e) x=0.20.
